# Parity, dental caries and implications for maternal depletion syndrome in northern Nigerian Hausa women

**DOI:** 10.1371/journal.pone.0281653

**Published:** 2023-03-02

**Authors:** Elizabeth O. Oziegbe, Lynne A. Schepartz

**Affiliations:** 1 Faculty of Dentistry, Obafemi Awolowo University, Ile-Ife, Nigeria; 2 Human Variation and Identification Research Unit (HVIRU), School of Anatomical Sciences, Faculty of Health Sciences, University of the Witwatersrand, Johannesburg, South Africa; 3 University of Pennsylvania Museum of Archaeology and Anthropology, Philadelphia, PA, United States of America; Shahid Beheshti University of Medical Sciences, School of Dentistry, ISLAMIC REPUBLIC OF IRAN

## Abstract

**Background:**

Female reproductive history, especially high parity, affects general health and may impact negatively on oral health. While parity has been positively linked to tooth loss, the specific association between parity and caries has not been adequately investigated.

**Aim:**

To determine the association between parity and caries in a population of higher parity women. Influences of likely confounders (age, socio-economic status, reproductive parameters, oral health practices and sugar consumption between meals) were considered.

**Methods:**

This was a cross-sectional study involving 635 Hausa women of varying parity aged 13–80 years. Socio-demographic status, oral health practices and sugar consumption were obtained using a structured interviewer-administered questionnaire. All decayed, missing and filled teeth due to caries (excluding third molars) were noted, and tooth loss etiology was queried. Associations with caries were evaluated through correlation, ANOVA, post hoc analyses and Student’s *t* tests. Effect sizes were considered for magnitude of differences. Multiple regression (binomial model) was used to investigate predictors of caries.

**Results:**

Hausa women had a high prevalence of caries (41.4%) despite low sugar consumption; nonetheless the overall mean DMFT score was very low (1.23 ± 2.42). Older, higher parity women experienced more caries, as did those with longer reproductive spans. Additionally, poor oral hygiene, use of fluoride toothpaste and frequency of sugar consumption were significantly associated with caries.

**Conclusion:**

Higher parity (>6 children) was associated with higher DMFT scores. These results suggest that a form of maternal depletion, expressed as heightened caries susceptibility and subsequent tooth loss, occurs with higher parity.

## Introduction

One largely neglected aspect of research on parity and its relationship to health is the oral health of mothers and their children. There is a general belief that women’s oral health declines with increasing numbers of children. Proverbs reflecting expected tooth loss due to pregnancy are known for many societies [1]. Previous research on parity and tooth loss in Nigerian Hausa women revealed that the cumulative demands of reproduction are associated with tooth loss in a complex manner involving the interplay of biological and behavioral factors [2]. In this study we investigate how dental caries, a key contributor to tooth loss, affects Hausa women of highly varying parity levels.

Tooth loss results from destruction of the tooth or surrounding oral tissues due to injury, infection, decay, inflammation or resorption. Of particular interest for this study are the processes relating to decay of the tooth structure and the loss of alveolar bone quality that culminate in the loss of teeth. The two major etiologies are dental caries and periodontitis; there is evidence suggesting that risks for both conditions are heightened in females, especially during the reproductive years [3, 4].

There are clear patterns of sex-based differences in oral health in many populations, especially for caries frequencies and, in some circumstances, tooth loss. Several studies identified a significant difference in the prevalence of dental caries in adults with higher levels in females in most populations. This bias toward poorer oral health in women is evident both in prehistoric and contemporary societies [3, 5, 6]. For example, in Bronze Age Mycenaeans from Pylos, Greece, the higher caries frequencies and tooth loss in females was correlated with stable isotope results indicating greater levels of cariogenic plant foods in female diets [6]. The data for contemporary populations collectively suggest that females tend to have more caries and that male-female differences in caries generally increase over the lifespan. The reason for the disparities has been attributed to earlier emergence of teeth in females with longer exposure to cariogenic oral environments, the influence of hormones and pregnancy, and the role of women in domestic food preparation and consumption [7]. Differential access to health care and varying attitudes toward dental treatment are additional factors that can result in sex-based oral health disparities [8].

Pregnancy and breastfeeding involve nutritional, physiological and metabolic adjustments that can cause permanent changes in stress responses. This is especially true when parity is high [9]. High parity (over four births) [10], together with short birth intervals, impacts negatively on health in terms of insufficient biological resources for the maintenance of maternal calcium reserves and oral tissues. While there is evidence for the normal recovery of maternal resources, the ability to return to pre-pregnancy levels may be compromised under certain conditions where subsequent pregnancies and limited resources co-occur [11]. The existence or exact nature of this phenomenon, termed Maternal Depletion Syndrome (MDS), remains contested although there is evidence for high parity effects on maternal health in some populations [12]. MDS has been linked to osteomalacia, which involves calcium depletion from bones and dental tissues with eventual loss of teeth [13]. More recent research identified altered cellular cementum in an osteomalacia patient, suggesting that the condition leads to disruption in cementogenesis, poor stability of the teeth and soft tissue inflammation [14].

Hormonal fluctuations and pregnancy have been argued to influence higher caries rates observed in females, although the exact mechanisms through which these two factors promote caries are not fully understood. Estrogen levels are known to increase significantly during menstruation and life-history events such as puberty and pregnancy [15]. Animal studies demonstrate that higher estrogen levels are associated with reduction in saliva flow rate and increased caries [16, 17].

Pregnancy is associated with extreme hormonal fluctuations. Estrogen levels are higher during pregnancy than for any other aspect in the female life cycle [18, 19]. During high estrogen phases the buffering capacity of saliva is reduced and this promotes growth of cariogenic bacteria [20]. Vomiting, neglected oral hygiene, cravings for refined carbohydrates and poor nutrition may also contribute to caries during pregnancy [21] and dental treatment is often curtailed or postponed for pregnant women [8]. Rakchanok *et al*. demonstrated that the odds of caries experience in Thai pregnant women were almost three times greater than non-pregnant women [22]. However, Mangi failed to demonstrate a link between pregnancy and caries in an Indian sample [23].

The available data on parity and caries are principally from clinical studies. Russell *et al*., using a large heterogeneous US sample, reported that women of high parity had more decayed and missing teeth due to caries compared to lower parity women who had more filled teeth due to caries [24]. However, Ueno *et al*. failed to establish a link between parity and decayed/filled teeth in a Japanese population [25]. Furthermore, studies from Africa [26–28] did not observe an association between parity and caries.

The association between parity and caries might not be transparent because most research on these conditions is cross-sectional or not specifically designed to focus on parity. Many studies fail to define high parity or to include high parity women and other reproductive parameters, or they do not consider confounding factors such as socio-demographic characteristics and oral health practices that may contribute to caries formation. Thus, it was important to assess the association between parity and caries among a high parity population of northern Nigerian Hausa women as part of a larger mixed methods community study on maternal and child oral health status [2, 29].

## Methods

### Study setting

The Hausa are the largest subnational ethnic group in West Africa. They are a diverse but culturally homogenous people living in northern Nigeria and southeastern Niger. The majority of Hausa live in the northwestern Nigerian region popularly referred to as “Hausaland”. In the villages, Hausa are primarily subsistence farmers who live together in a simple feudal system under one leader (emir) [30]. Their traditional culture is guided by laws and social behaviors based on Islamic teachings [31]. This includes the emphasis placed on large family size.

### Study location

The study was conducted in Kumbotso Local Government Area (LGA) of Kano State, Nigeria. Kano State is located in the northwest zone of Nigeria and has a population of 9.4 million [32]. Kumbotso LGA has a population of 295,979 and spans an area of 158 km^2^. According to the 2006 census, 66,010 women aged 15–65 years reside in Kumbotso LGA.

### Sample population

The sample population was selected through a household survey in the Kumbotso LGA using a multi-stage random sampling technique. The LGA comprises of 11 administrative wards. Each of the wards was assigned a number and then six numbers were drawn at random to choose the selected wards for the study. Within each ward, two communities were selected using a random sampling technique and all households in each community were approached. All women in the households (including widowed and divorced women) who met the inclusion criteria of good general health and age between 13 and 80 years were interviewed and examined. The minimum age of 13 years was used as it is the mean female age at marriage [33]. The upper age limit was set to 80 years to capture the cumulative effects of parity. Women with severe medical conditions, or conditions associated with xerostomia (dry mouth) such as diabetes and Sjogren’s disease, as well as those who had been on antibiotics three to four weeks prior to oral examination, were excluded from the study. The exclusion of those on antibiotics was necessary for the larger study that assessed the periodontal health of the participants.

### Group composition

Women of all parity levels were included in the study. The parity levels were separated into two basic groups: low parity women who had given birth less than five times regardless of whether the child survived, and high parity women who had 5 or more births. The upper cut point of ≥5 births was selected as it is the level where health risks and morbidity differences are seen. It is also the accepted definition of high parity [10].

### Data collection method

A structured interviewer-administered questionnaire (S1 File) was used to obtain information on the socio-demographic status and oral health practices of the women. Section A captured information on age, educational status (none, primary, secondary, Koranic or tertiary), occupation of the woman, age at first pregnancy and the number of children the women had given birth to as well as intervals between births. Section B elicited information on the oral health behaviors and diet. The questions covered frequency of tooth brushing (I don’t brush, once daily, twice daily and >twice daily), regular use of fluoridated toothpaste and frequency of dental visits. Information on frequency of daily consumption of refined carbohydrates between meals was collected to access dietary risk factors in light of the Vipeholm study that documented individuals who frequently consumed refined sugar between meals had significantly increased caries experience [34].

#### Socio-economic status

The socio-economic status was scored using the Standard Occupation Classification designed by the Office of Population Census and Surveys [35]. The occupations were grouped into the following classes:

Social class I: professional occupations, Social class II: managerial and technical occupations, Social class III (NM): skilled occupations (non manual), Social class III (M): skilled occupations (manual), Social class IV: partly skilled occupations and Social class V: unskilled. The social classes I and II were grouped as high socio-economic status, classes III (NM) and III (M) as middle, and classes IV and V as low socio-economic status.

Determination of socio-economic status for the women was complicated by the multi-household structure of families. For this reason, we focused on women;s work alone for the scoring.

#### Intra oral examination

Intra oral examination was carried out using a sterile dental mirror and probe with the participant seated on a chair in a well-lit environment. All the teeth present in the mouth, with the exclusion of the third molars, were recorded using the Fédération Dentaire Internationale (FDI) notation. The DMFT index of the World Health Organization for dental epidemiological studies was used to determine the caries status of the participants [36]. The index includes the following: D- number of decayed teeth, M- number of extracted teeth due to caries, F- number of teeth filled or crowned due to caries and T- teeth present. Only teeth extracted due to caries were recorded as missing. Histories of missing teeth was elicited from the participants in order to exclude agenesis, trauma and other causes. Women who were caries free would have DMFT scores of 0. ‘Caries experience’ was denoted by any circumstance where where the DMFT score was ≥1. Oral hygiene status was evaluated using the Simplified Oral Hygiene Index (OHI-S) [37]. The index comprises of debris and calculus scores on selected tooth surfaces. The buccal and lingual surfaces of the six index permanent teeth (FDI numbers 11, 16, 26, 31, 36, 46) were examined. Oral hygiene was classified as *good*, *fair*, or *poor* when score ranges were 0.0–1.2, 1.3–3.0, and >3.0, respectively.

### Ethical considerations

Ethical clearance for the study was obtained from the Ethics and Research Committee of Obafemi Awolowo University, Ile-Ife, Osun State, Nigeria (IPHOAU/12/717) and the Health Sciences Research Ethics Committee of the University of the Witwatersrand, Johannesburg, South Africa (M170343). Permission to conduct the study was obtained from the local village leaders. A male local assistant who spoke Hausa fluently was employed to facilitate links with village leaders and husbands of the women. All of the mothers were formally married according to Sharia law and living in the home of their husbands and not their family of origin. Informed consent was obtained from the husbands of married women living with their husbands. Married women are deemed able to give informed consent in the context of the Hausa traditional society and we respected that in conducting our research. Written informed consent was obtained from each participant. The consent form was translated into Hausa and read to participants who were illiterate; those who were then willing to participate in the study thumb printed on the consent form. Only women who were willing to participate and gave informed consent were included.

### Data analysis

The data were analyzed using SPSS (version 16) software for Windows. Analysis included all women at the different levels of parity. Descriptive analyses of age, socio-economic status, parity, behavioral factors (such as frequency of tooth brushing per day, use of fluoridated toothpaste and consumption of refined sugar) were done. The mean DMFT scores of the different levels of parity, and between low and high parity groups, were compared using ANOVA and Student’s *t* tests respectively. Correlation analysis was done between parity and mean DMFT scores. Lastly, the effect of predictors on caries was explored. A Shapiro-Wilk test showed a departure of the data from normality (W = 0.55, p = 0.00). Therefore multiple logistic regression was used to evaluate the association between the independent variables (socio-demographic, reproductive parameters and oral hygiene practice/behavior and the outcome caries experience as expressed by the DMFT score. Ten independent variables fitted in the model included age, age at first birth, age at last birth, parity, duration of reproduction, SES, oral hygiene status, frequency of tooth cleaning, fluoride in toothpaste and frequency of refined sugar consumption. Statistical significance was inferred at p<0.05.

## Results

### Socio-demographic characteristics of participants

Six hundred and thirty-five women participated in the study. Their ages ranged from 13 to 80 years with a mean age of 32.19 ± 12.72 years. The majority (65.1%) of the participants were between the ages of 18 and 37 years. The extremes of the age cohorts had the least number of participants, with 5.4% aged 13–17 years and 1.7% aged ≥66 years (S1 Table in S1 File).

Approximately 5% of the participants were illiterate. The level of general education was low with 22% of women having attended primary school and 0.5% with some tertiary education. More than 50% of the women had the form of education referred to as *Islamiyyah* schooling where the emphasis is on Koranic learning (S1 Table in S1 File).

Determination of socio-economic status for the women was complicated by the multi-household structure of families. For this reason we focused on women’s work alone for the scoring. According to their individual status, the majority (64.9%) of the participants were of middle socio-economic status and supported themselves working as market traders of cereal grains and legumes (soya beans, millet and other beans). The remaining 35.1% were housewives of low socio-economic status with no outside employment. There were no women of high socio-economic status (S1 Table in S1 File).

#### Reproductive parameters of participants

About 8% of the women were nulliparous, with the majority of these (83%) in the youngest age cohort. The remaining childless women were equally distributed between the age groups 28–37 years and above 47 years (8.5% in each). As expected, parity varied proportionally to increasing age in the sample. The highest parity of 17 children was observed for a woman in the age cohort 58–65 years. There were increasing proportions of women with more than four children between the ages of 28 and 47 years. Above 37 years, few women had less than four children or greater than 10 children. Thirty-one women (4.9%) had ten or more children and they were aged between 28 and 65 years (S2 Table in S1 File).

There were more women in the low parity group (≤4 children) compared to the high parity group (≥5 children) (55.7% and 44.3% respectively). The overall mean parity was 4.33 ± 3.04 children per woman or just below the standard definition of high parity. Considering mean parity by age cohorts, the 34 participants aged 13–17 years had the lowest mean parity of 0.68 **±** 0.91 while the highest mean parity (7.21 **±** 3.70) characterized the 24 women aged between 58 and 65 years (S2 Table in S1 File).

The mean age at first birth (17.59 **±** 3.45) was relatively young. The majority of the participants (72.2%) had their first child before 19 years of age. Half of those aged 13–17 years had already given birth, and one had four children before age 18. The mean age at last birth (27.73 **±** 7.88), while less informative as many participants had not completed their reproductive years, suggests that most women were reproductively active for periods of 20 years or more.

Other reproductive parameters that provide information relating to maternal health condition are the mean birth interval (24.11 **±** 8.52 months) and the duration of breastfeeding (19.78 **±** 2.77 months). The birth intervals were quite variable, yet the mean of under two years was short given that breastfeeding continues for at least one year and up to 5 years (mean of 19.78 **±** 2.77 months). These data indicate that many women were breastfeeding one child while gestating another. These reproductive characteristics reflect the predominance of younger women in the sample as well as the importance of pronatalism in the traditions of the Hausa people (S3 Table in S1 File).

#### Relationship between parity and caries experience, including the investigation of possible confounders

Caries experience (presence/absence) among the participants had an overall prevalence of 41.4%. The caries experience differed by parity with more caries-free women observed in the low parity levels. Among these, women with only one child were the most caries-free (82%). Interestingly, at parity 5, there was no difference in the proportion of women who were caries-free and caries-active (both at 50%). However, from parity 6 and above, the frequency of caries experience was higher, with the exception of parity 8. Women with nine or more children had the highest proportion of caries presence (60.9%) (**Table 1**).

**Table 1:** Frequency of caries by parity and parity groups.

Parity	N	Caries absent	Caries present
		N (%)	N (%)
0	47	30 (63.8)	17 (36.2)
1	89	73 (82.0)	16 (18.0)
2	69	44 (63.8)	25 (36.2)
3	74	50 (67.8)	24 (36.2)
4	75	46 (61.3)	29 (38.7)
5	82	41 (50.0)	41 (50.0)
6	43	20 (46.4)	23 (53.5)
7	54	22 (40.7)	32 (51.3)
8	38	21 (55.3)	17 (44.7)
≥9	64	25 (39.1)	39 (60.9)
**Parity group**			
Low	354	243 (68.6)	111 (45.9)
High	281	129 (45.9)	152 (54.1)
**Total**	**635**	**372 (58.6)**	**263 (41.4)**

These general descriptive results for caries presence/absence suggest that the caries experience of the women was potentially influenced by parity. To verify this, it is necessary to examine caries and parity in greater detail using DMFT scores and controlling for age and other potential confounders.

DMFT scores provide more information regarding differences in caries severity. The scores ranged from 0 to 26 with an overall mean value of 1.23 ± 2.42., This overall mean DMFT score (**Table 2**) was very low according to the WHO standard of mean DMFT index (very low <5.0, low 5.0–8.9, moderate 9.0–13.9 and high >13.9) [38]. Although some women had individual scores that were extremely high, there was considerable variation as indicated by the large standard deviations (**Table 2**). Table 2 also shows the relationship between reproductive parameters (parity group, age at first birth, duration of reproduction and interbirth intervals) and mean DMFT score. Parity group (low vs. high) was found to be significantly associated with caries experience (p = 0.00) using the Student’s *t* test analysis: 95% CI (-1.36, -0.62) with a small effect size of 0.4. The mean DMFT score for the high parity group was significantly higher than the low parity group (**Table 2**). Age at first birth was not associated with the mean DMFT score. The scores differ considerably across the groups but there was no consistent pattern, and the standard deviations were large except for those women first giving birth at age 30 and older.

**Table 2:** Mean DMFT scores by reproductive parameters.

Covariates	N	Mean ± SD	Significance
**Parity group**			
Low parity ≤4^a^	354	0.79 **±** 1.56	t = -5.25
High parity ≥5^b^	281	1.78 **±** 3.09	p = 0.00*
**Age at first birth**			
≤18	431	1.21 **±** 2.45	F = 0.76
19–24	133	1.44 **±** 2.66	p = 0.56
25–29	18	1.17 **±** 1.79	
≥30	15	0.40 **±** 0.63	
**Duration of reproduction**			
≤5^c^	222	0.61 **±** 1.27	F = 14.93
6–11^d^	158	0.95 **±** 1.47	p = 0.00**
12–17^e^	126	1.63 **±** 3.08	
≥18^f^	129	2.22 **±** 3.51	
**Inter birth interval**			
<3	505	1.12 **±** 2.23	F = 2.42
3–5	112	1.61 **±** 3.05	p = 0.90
≥6	18	1.78 **±** 2.77	
**Overall mean**	635	1.23 **±** 2.42	

*Student’s *t* test Parity group: ^a,b^95% CI [-1.36, -0.62]; p 0.00; SDM = 0.4.

**Post hoc analysis Duration of reproduction: ^c,e^95% CI [-1.48, -0.56]; p = 0.00; SDM = 0.5. ^c,f^95% CI [-2.12, -1.10]; p = 0.00; SDM = 0.7. ^d,f^95% CI [-1.88, -0.66]; p = 0.00; SDM = 0.5.

The mean DMFT score varied with increasing duration of reproductive years. Women reproductively active for ≤5 years had the lowest caries experience of 0.61 ± 1.27, while those active 18 years or more had the highest mean score (2.22 ± 3.51). The overall trend was similar for interbirth intervals. Post hoc analysis with Games-Howell multiple comparisons of means revealed a statistically significant difference between mean DMFT scores and duration of reproduction (F = 14.93, p = 0.00) but not between mean DMFT scores and interbirth intervals (**Table 2**). Women with reproductive years of ≤5 had significantly lower mean DMFT scores than those of 12–17 years (0.61 ± 1.27 vs. 1.63 ± 3.08), p = 0.00, 95% CI (-1.48, -0.56), standard deviation of the means (SDM) = 0.5. While participants with ≥18 years of reproductive activity experienced significantly higher mean DMFT score than those of ≤5 years activity (2.22 ± 3.51 vs. 0.61 ± 1.27), p = 0.00, 95% CI (-2.12, -1.10) with a medium effect size of 0.7. Similarly, women with the longest reproductive span (≥18 years) had significantly higher mean DMFT scores compared to those with a span of 6–11 years (2.22 ± 3.51 vs. 0.95 ± 1.47), p = 0.00, 95% CI (-1.88, -0.66), SDM = 0.5. These results were expected because the duration of reproduction is very much related to age.

Women with high parity experienced more caries than those with low parity in all age cohorts except 18–27 years. However, this difference was not statistically significant (p>0.05) based on the Student’s *t* test analysis (**Table 3**), and was due to the wide standard deviations. The mean DMFT score varied proportionally with increasing age in both parity groups, but the trend was most clear for the high parity group (**Table 3**). The highest mean DMFT score for that group was 3.17 ± 2.93 observed in women aged ≥66 years, while in the low parity group it was 2.80 ± 2.77 for the same age group. The lowest mean DMFT scores for the parity groups were 0.46 ± 0.86 and 0.50 ± 1.05, observed in high parity women aged 18–27 and low parity 13–17 year olds respectively.

**Table 3:** Mean DMFT scores by parity group.

Age group (years)	Low parity ≤4		High parity ≥5		p-value	95.0% Confidence Interval		SDM
	N (354)	Mean ± SD	N (281)	Mean ± SD		Lower Bound	Upper Bound	
13–17	34	0.50 **±** 1.05	0	**-**	**-**	**-**	**-**	**-**
18–27	210	0.54 **±** 1.13	26	0.46 **±** 0.86	0.73	-0.37	0.53	0.1
28–37	69	1.09 **±** 1.94	108	1.45 **±** 2.65	0.33	-1.09	0.37	0.2
38–47	24	1.71 **±** 2.68	77	1.75 **±** 2.37	0.94	-1.17	1.09	0.02
48–57	9	1.44 **±** 1.88	43	2.67 **±** 4.06	0.38	-4.03	1.57	0.3
58–65	3	1.67 **±** 2.08	21	3.00 **±** 5.70	0.70	-8.33	5.67	0.2
≥66	5	2.80 **±** 2.77	6	3.17 **±** 2.93	0.84	-4.29	3.55	0.1

Mean DMFT scores differed by parity level. Women with only one child had the lowest mean score of 0.33 while those with nine or more children had the highest score of 2.92. There was an apparent trend of increase in mean DMFT score from parity 5 and above, although there was a reduction for the smaller sample of parity 8. The correlation between parity and mean DMFT score was statistically significant using a Spearman’s Rho non-parametric test (ρ = 0.81, p = 0.01). Parity was positively correlated with caries experience in Hausa women (S4 Table in S1 File).

### Association between socio-demographic variables and mean DMFT scores

Participant caries experience varied by age, socio-economic status and level of education. Post hoc analysis (Games-Howell multiple comparisons of means) showed that the mean DMFT scores differed significantly by age cohorts (F = 10.37, p = 0.00), with the variation most characteristic of the extremes of the age groups (13–17 vs. ≥66 years). Large effect sizes were observed in the mean DMFT between age groups 13–17 years, 18–27 years, 38–47 years, 48–57 years, 58–65 years, and those 66 years and above (1.6, 0.8, 1.0, 1.2 and 2.0 respectively). However, the mean DMFT scores were not significantly associated with socio-economic status or level of education using Student’s *t* test (p>0.05 respectively). This reflects the homogenous character of the sample population; there was little difference in the SES and educational attainment levels that could impact caries experience (S5 Table in S1 File).

### Association between oral health and mean DMFT scores

**Table 4** provides information on the associations between mean DMFT scores and the covariates of oral hygiene status and oral health practices. According to the post hoc analysis, oral hygiene status was significantly associated with the women’s caries experience. Women with poor oral hygiene had the highest DMFT scores while those with good oral hygiene had the lowest. The significant difference observed was among the three categories of oral hygiene status (ac and bc) with a medium effect size of SDM = 0.5 observed between the extremes of oral hygiene status. Women who visited the dentist had significantly higher caries experience than those who did not seek dental treatment based on Student’s *t* test analysis (2.18 ± 3.42 vs. 1.15 ± 2.29 respectively), suggesting that women with symptomatic carious teeth were more likely to seek dental care. The remaining covariates (frequency of sugar consumption between meals, tooth cleaning and fluoride in toothpaste) were not significantly associated with caries experience (p>0.05) **(Table 4)**.

**Table 4:** Mean DMFT scores by oral hygiene status and practices/behaviors.

Covariates	N	Mean ± SD	Significance
**Oral hygiene status**			
Good^a^	16	0.13 ± 0.34	F = 7.75
Fair^b^	195	0.76 ± 1.46	p = 0.00*
Poor^c^	424	1.48 ± 2.75	
**Frequency of tooth brushing**			
I don’t brush	12	1.58 ± 2.50	F = 1.05
Once daily	77	1.51 ± 2.59	p = 0.37
Twice a day	213	1.01 ± 1.65	
>Twice a day	333	1.29 ± 2.76	
**Fluoride in toothpaste**			
Yes	393	1.09 ± 2.07	t = -1.83
No	242	1.45 ± 2.88	p = 0.07
**Visit to the dentist**			
Yes^d^	49	2.18 ± 3.42	t = 2.09
No^e^	586	1.15 ± 2.29	p = 0.00**
**Frequency of sugar consumption between meals**			
Always	61	1.70 ± 3.32	F = 1.78
Sometimes	303	1.27 ± 2.47	p = 0.17
Rarely	271	1.07 ± 2.09	

*Post hoc analysis- Oral hygiene status: ^a,c^95% CI [-2.70, 0.00]; p = 0.04; SDM = 0.5. ^b,c^95% CI [- 1.13, -0.31]; p = 0.00; SDM = 0.3.

**Student’s *t* test- Visit to the dentist: ^d,e^95% CI [0.33, 1.73]; p = 0.00; SDM = -0.4.

### Multivariate analysis of predictors of caries experience

A generalized linear model (binomial) was used to predict variables associated with the DMFT score. Women’s age, parity, age at first birth, duration of reproduction, oral hygiene status, fluoride in toothpaste and frequency of consumption of refined sugar were significantly associated with the DMFT score (p<0.05 respectively) (**Table 5**).

**Table 5:** Multiple regression for predictors of DMFT.

Predictors	Odds ratio**	SE	z	P	95.0% Confidence Interval	
					Lower Bound	Upper Bound
Age	1.02	0.00	7.50	0.00*	1.02	1.03
Age at first birth	1.00	0.00	3.19	0.00*	1.00	1.09
Age at last birth	0.99	0.00	-1.05	0.30	0.99	1.00
Parity	1.06	0.02	3.74	0.00*	1.03	1.10
Duration of reproduction	1.15	0.05	3.07	0.00*	1.05	1.26
Low socio-economic status	1.10	0.09	1.25	0.21	0.95	1.29
Fair oral hygiene status	5.15	3.68	2.29	0.02*	1.27	20.90
Poor oral hygiene status	6.92	4.94	2.71	0.07*	1.71	20.02
Once daily tooth brushing	1.24	0.31	0.86	0.39	0.76	2.03
Twice daily tooth brushing	1.19	0.29	0.70	0.48	0.73	1.93
More than twice daily tooth brushing	1.38	0.33	1.35	0.18	0.86	2.23
Fluoride absent	1.19	0.09	2.30	0.02*	1.03	1.38
Sometimes consumes refined sugar between meals	0.77	0.09	-2.34	0.02*	0.62	0.96
Rarely consumes refined sugar between meals	0.54	0.06	-5.25	0.00*	0.43	0.68
(Constant)	0.04	0.03	-4.09	0.00	0.01	0.20

Deviance = 1538.608227; (1/df) Deviance = 2.481626; Pearson = 1944.251318; (1/df) Pearson = 3.135889; Akaike Information Criterion (AIC) = 3.600465

* Significant values

**The odds ratio is between two groups of the categorical variables; for the continuous variables (age, age at first and last birth and duration of reproduction), it is with respect to increase in age by one year.

## Discussion

Our study provides the first evidence that parity is positively associated with caries severity in an African population. Higher parity women had significantly more caries than lower parity women, irrespective of their socio-economic status and oral health practices.

As caries severity and parity are largely time-dependent measures, it is not surprising that increased age was associated with higher DMFT scores, and that women of higher parity experienced more severe tooth decay. The older an individual is, the greater potential exposure of teeth to the oral environment and cariogenic factors (primarily refined sugar and *Streptococcus mutans*) that favor caries formation. However, age did not fully explain the relationship between caries and parity in Hausa women. While age influences both caries and parity, parity still contributed significantly to caries severity in the Hausa women. Their caries experiences were related to their reproductive history—specifically their age at first birth, parity and duration of reproductive years—in conjunction with caries risk behaviors.

While most Hausa women’s caries risk behaviors did not include extreme sugar consumption, the frequency of sugar consumption between meals was significantly associated with caries. This follows the expected pattern documented in other populations. Women who “always” consumed sugar had higher DMFT scores than those who”sometimes” or “rarely” consumed sugar. The other behaviors evaluated as risks for caries, specifically oral hygiene status and use of fluoridated toothpaste, also were positively associated with caries experience and higher DMFT scores.

The women in our study had fairly similar levels of dental health care access and usage. Use of treatment services was very low for both low and middle-income women and reflected similar behaviors involving delayed treatment of caries with extraction as the typical outcome. We observed that the caries experience was not associated with SES. This reflects the homogenous character of our sample where SES differentials are not substantial. What differences do exist are mostly age-related as older Hausa women are much more likely to be involved in work or business enterprises outside the home. In other contexts, socio-economic status has been argued to be a key contributor to women’s oral health status and related tooth loss, with individuals of low SES having the poorest oral health [39, 40], but this is not the case for Hausa women in our study.

### Caries and African populations

Caries frequencies in African populations, as well as DMFT scores, are typically low compared to other world regions [38, 41]. This is unexpected given the low oral health awareness levels and poor use of preventative measures in many areas of Africa [38]. However, dietary factors potentially underlie these different caries experiences. The refined sugar consumption of Hausa women was low, and this may best explain their comparatively low caries incidence and DMFT scores. Indigenous rural African diets do not support caries formation as they are primarily comprised of unprocessed ingredients that are low in refined sugars [42]. The Hausa diet consists of grains such as millets that are rich in starch. Presently, the role of high starch diets in caries formation is debated, although it has been documented that salivary enzymes convert the starch in foods to glucose in very small amounts [42]. There is evidence suggesting that starch-rich dietary staples are associated with low caries experience, particularly in diets where refined sugar consumption is also low [43, 44]. The high starch diet and caries relationship appears to be more complex than originally thought, with more research needed on relative proportions of starch-rich staples and other foodstuffs, as well as patterns of consumption [45]. In addition, hard and coarse diets can modify the occlusal morphology of molars in ways that can alter caries exposure. The attritional effects would be more pronounced for teeth with low surface relief and thin enamel, although any loss of occlusal surface features such as deep grooves would potentially reduce caries risk. Enamel thickness is associated with the caries process, with thicker enamel being less susceptible to caries [46]. Americans of African ancestry were found to have thicker enamel than those of European ancestry [46, 47], implying they might have greater caries resistance. Further research is needed on enamel thickness in African populations and how it figures into the reported low prevalences of caries in Africans, particularly those subsisting on more traditional diets with limited processed foods [41].

### Other African studies on parity and caries

Three previous studies on African women did not identify any relationship between parity and caries. Walker *et al*. observed no difference in the caries rates of higher and lower parity rural Black South Africa women [26]. Similarly, Scheutz *et al*. failed to detect an association between parity and caries in Tanzanian women [27]. The differences between those studies and ours are likely to be due to the sample demographics. Women older than 50 years and 55 years were excluded from the Tanzanian and South African studies respectively. Similar to the situation with tooth loss, the cumulative effects of high parity on caries are likely to be more obvious in the older age groups. Consequently, the results of these two studies may not be a true reflection of the relationship between parity and caries in African women. A recent study among a group of nursing mothers from Southeast Nigeria failed to observe an association between parity and caries [28]. In this case, the difference observed may be related to the research design, which was a hospital-based study with few higher parity women and exclusion of women older than 52 years.

### Caries, parity and maternal depletion

Our study determined that higher parity women experienced significantly more caries than women of lower parity. This supports the argument that there is decreased resistance to caries in higher parity due to the cumulative effects of heightened hormone levels, longer reproductive spans and short birth intervals. Increased estrogen during pregnancy provides a favorable environment for caries [20]. In addition, there is hyperactivity of the gingivae leading to gingivitis and subsequently periodontitis, although these soft tissue effects are potentially reversible [48]. Compounding over successive pregnancies, these conditions may be major contributory factors for more caries and periodontitis in higher parity women, with eventual tooth loss if no dental care takes place. Indeed, higher parity women in our study experienced more tooth loss, even though severe periodontitis was rare among the participants [2]. These results are indicative of depletion of maternal biological resources for maintaining good oral health in higher parity women.

### Policy implications

Pregnancy and maternity are known to change dental care utilization patterns [8]. The Hausa women had poor utilization of oral health care services. Similarly, few nursing mothers from Southeast Nigeria visited the dentist [28]. In Nigeria, utilization of dental care service is poor, due to limited availability and accessibility of oral health clinics. Thus the underlying reasons for untreated caries observed in our sample will eventually contribute to more tooth loss. In addition, dental health problems are perceived as not life threatening, and so low priority is given to dental care relative to other health seeking behaviors [49]. There is a need for oral health awareness and education among the Hausa women to promote attitudinal changes and to identify barriers to dental care service. On a broader scope, this study highlights the need to monitor oral health conditions as part of pregnancy support for women in developing countries, with particularly focus on those of higher parity.

### Limitations

A cause and effect relationship cannot be inferred between parity and caries due to the cross-sectional design of this study. Establishing a direct link between MDS and caries would require detailed assessment of nutritional status and caries experience over several phases of the life course. Thus a longitudinal study is needed to verify a causal effect of MDS in caries experience.

## Conclusion

This study demonstrated that reproductive parameters (age at first birth, duration of reproduction and parity) were positively associated with caries experience. The prevalence of caries among Hausa women was fairly high, even though the mean DMFT score was very low. Women with a longer span of reproductive years and higher parity experienced more caries. As expected, age was significantly associated with caries severity as determined by higher DMFT scores. In addition, poor oral hygiene, limited use of fluoridated toothpaste and higher frequency of sugar consumption were significantly associated with greater severity of caries. These results are indicative of some level of maternal biological depletion in higher parity Hausa women.

## Supporting information

S1 File Data collection form and S1-S5 Tables. (DOC)

S1 Data Oziegbe Schepartz caries data. (XLSX)
