# Supplementary material for: Parity, dental caries and implications for maternal depletion syndrome in northern Nigerian Hausa women
Source: PLoS One. 2023 Mar 2;18(3):e0281653. doi: 10.1371/journal.pone.0281653 (PMC9980799; doi:10.1371/journal.pone.0281653)
Supplement: S1 File — (DOC) [file pone.0281653.s001.doc]

**S1 Data collection form and Tables S1-S5**

**Data collection form**

Section A

1. Serial number:

2. Sex: Female

3. Age: 4. Date of birth

5. Ethnic group:

6. Type of marriage: (a) Monogamous (b) Polygamous (Number of wives……..)

7. (a) Age at first child: (b) Age at last child:

8. Number of pregnancies:

9. (a) Number of children: (b) Age between each child:

10. Duration of breastfeeding per child:

11. (a) Occupation ………………(b) Occupation (husband)…………………………

12. Level of education (a) None (b) Primary (c) Secondary (d) Koranic (e) Tertiary

Section B

13. Is there any special food you eat a lot during pregnancy? (a) Yes (b) No

14. If yes to question 12, what is/are the special food(s)?

------------------------------

------------------------------

------------------------------

15. How often do you consume sugar containing snacks or drinks between your main meals?

(a) Three or more times a day (b) Twice a day (c) Once a day (d) Occasionally not everyday (e) Rarely or never eat between meals (e) No response

16. How often do you usually brush your teeth?

(a) Irregularly or never (b) Once a week (c) Two to three times a week (d) Once a day (e) Twice a day (f) more than twice a day (f) No response.

17. When do you clean your teeth?

(a) Morning before breakfast (b) Morning after breakfast (c) Afternoon before lunch (d) Afternoon after lunch (e) Evening before supper (f) Evening after supper (g) No regular time interval (h) No response.

18. What do you use to clean your teeth?

(a) Tooth brush and toothpaste (b) Chewing stick (c) Hand and water (d) Hand and salt (e) Cotton wool and salt (f) Others (specify)…………………..

19. Do you use toothpaste to clean your teeth? (a) Yes (b) No

20. If yes to question 19, what is the name of your toothpaste ……………..

21. (a) Toothpaste contains fluoride (b) Toothpaste does not contain fluoride (to be answered) by the PI

22. How often do you use fluoride-containing toothpaste?

(a) Always (b) Sometimes (c) Rarely (d) Never

23. When you have problems with your teeth where do you go …………

24. When last did you visit the dentist?

(a) Last 6 months (b) more than 6 months to 1 year ago (c) 1 to 2 years ago (d) 2 to 5 years ago (e) More than 5 years ago (f) Never (g) No response

25. When you visited the dentist, what treatment was carried out?

(a) Extraction of teeth (b) Filling of a hole on a tooth (c) Cleaning of my teeth (d) Others (specify)………….

26. Dental caries status using DMFT: (Put D on Decayed, M on missing teeth due to caries and F on filled teeth.)

Section C (oral examination)


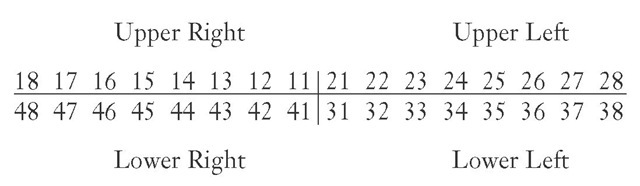


27. Oral hygiene status.

a) Good (b) fair (c) poor

Table S1 Socio-demographic characteristics of participants

| **Age (years)** | **N (%)**  **(Total N=635)** |
| --- | --- |
| 13-17 | 34 (5.4%) |
| 18-27 | 236 (37.2%) |
| 28-37 | 177 (27.9%) |
| 38-47 | 100 (15.7%) |
| 48-57 | 53 (8.3%) |
| 58-65 | 24 (3.8%) |
| ≥66 | 11 (1.7%) |
| **Level of education** |  |
| None | 30 (4.7%) |
| Koranic | 358 (56.4%) |
| Primary school (partial or completed) | 139 (21.9%) |
| Secondary school (partial or completed) | 105 (16.5%) |
| Tertiary (partial or completed) | 3 (0.5%) |
| **Socio-economic status (SES)** |  |
| High (Class I) | 0 (0.0%) |
| Middle (Class II) | 412 (64.9%) |
| Low (Class III) | 223 (35.1%) |

Table S2 Parity by age cohort

| **Age (years)** | **Parity** | | | | | | | | | | | | | | | | | **Mean ± SD** | **Total** |
| --- | --- | --- | --- | --- | --- | --- | --- | --- | --- | --- | --- | --- | --- | --- | --- | --- | --- | --- | --- |
|  | **0** | **1** | **2** | **3** | **4** | **5** | **6** | **7** | **8** | **9** | **10** | **11** | **12** | **13** | **14** | **15** | **17** |  |  |
| 13-17 | 17 | 14 | 1 | 1 | 1 | 0 | 0 | 0 | 0 | 0 | 0 | 0 | 0 | 0 | 0 | 0 | 0 | 0.68 ± 0.91 | **34** |
| 18-27 | 22 | 65 | 46 | 48 | 29 | 20 | 3 | 3 | 0 | 0 | 0 | 0 | 0 | 0 | 0 | 0 | 0 | 2.36 ± 1.59 | **236** |
| 28-37 | 4 | 9 | 15 | 13 | 28 | 34 | 19 | 21 | 18 | 9 | 5 | 1 | 1 | 0 | 0 | 0 | 0 | 5.19 ± 2.47 | **177** |
| 38-47 | 0 | 0 | 6 | 5 | 12 | 11 | 10 | 20 | 11 | 16 | 4 | 1 | 2 | 1 | 0 | 1 | 0 | 6.65 ± 2.58 | **100** |
| 48-57 | 1 | 1 | 1 | 4 | 3 | 10 | 5 | 7 | 5 | 7 | 4 | 1 | 2 | 2 | 0 | 0 | 0 | 6.79 ± 2.94 | **53** |
| 58-65 | 1 | 0 | 0 | 1 | 1 | 6 | 4 | 3 | 1 | 1 | 2 | 1 | 1 | 0 | 1 | 0 | 1 | 7.21 ± 3.70 | **24** |
| ≥66 | 2 | 0 | 0 | 2 | 1 | 1 | 2 | 0 | 3 | 0 | 0 | 0 | 0 | 0 | 0 | 0 | 0 | 4.64 ± 2.94 | **11** |
| **Total** | **47** | **89** | **69** | **74** | **75** | **82** | **43** | **54** | **38** | **33** | **15** | **4** | **6** | **3** | **1** | **1** | **1** | **4.33** ± **3.04** | **635** |

Table S3 Descriptive statistics: reproductive parameters

| **Reproductive parameters** | **N** | **Mean ± SD** | **Range** |
| --- | --- | --- | --- |
| Parity | 635 | 4.33 **±** 3.04 | 0-17 |
| Age at first birth (years) | 576 | 17.59 **±** 3.45 | 11-40 |
| Age at last birth (years) | 575 | 27.73 **±** 7.88 | 13-50 |
| Birth intervals (months) | 478 | 24.11 **±** 8.52 | 12-108 |
| Duration of breastfeeding (months) | 548 | 19.78 **±** 2.77 | 12-60 |

Table S4 Correlation analysis between parity and mean DMFT score

| **Parity** | **N** | **Mean DMFT**  **score** |  |
| --- | --- | --- | --- |
| 0 | 47 | 0.97 | ρ=0.81 |
| 1 | 89 | 0.33 | p=0.01* |
| 2 | 69 | 0.99 |  |
| 3 | 74 | 0.84 |  |
| 4 | 75 | 0.97 |  |
| 5 | 82 | 1.34 |  |
| 6 | 43 | 1.33 |  |
| 7 | 54 | 1.96 |  |
| 8 | 38 | 1.08 |  |
| ≥9 | 64 | 2.92 |  |

Table S5 Mean DMFT scores by age cohort, education level and SES

| **Covariates** | **N** | **Mean** ± **SD** | **Significance** |
| --- | --- | --- | --- |
| **Age group** |  |  |  |
| 13-17^a^ | 34 | 0.50 ± 1.05 | F=10.37 |
| 18-27^b^ | 236 | 0.53 ± 1.10 | p=0.00* |
| 28-37^c^ | 177 | 1.31 ± 2.37 |  |
| 38-47^d^ | 100 | 1.76 ± 2.42 |  |
| 48-57^e^ | 53 | 2.42 ± 3.77 |  |
| 58-65^f^ | 24 | 2.83 ± 5.37 |  |
| ≥66^g^ | 11 | 3.00 ± 2.72 |  |
| **Level of education** |  |  |  |
| None | 30 | 1.37 ± 2.17 | F=1.42 |
| Koranic | 358 | 1.36 ± 2.54 | p=0.21 |
| Primary | 139 | 1.27 ± 2.77 |  |
| Secondary | 105 | 0.68 ± 1.19 |  |
| Tertiary | 1 | 1.33 ± 2.31 |  |
| **SES** |  |  |  |
| Middle | 412 | 1.28 ± 2.41 | t=0.75 |
| Low | 223 | 1.13 ± 2.42 | p=0.46 |

Post hoc analysis Age group: ^a,e^ 95% CI [-3.24, -0.60]; p = 0.00; SDM = 0.6.

^a,f^ 95% CI [-4.22, -0.44]; p = 0.02; SDM = 0.7. ^a,g^95% CI [-3.62, -1.38]; p = 0.00; SDM = 1.6.

^b,c^ 95% CI [-1.12, -0.44]; p = 0.00; SDM = 0.4. ^b,d^ 95% CI [-1.61, -0.85]; p = 0.00; SDM = 0.8.

^b,e^ 95% CI [-2.45, -1.33]; p = 0.00; SDM = 1. ^b,f^ 95% CI [-3.11, -1.49]; p = 0.00; SDM = 1.2.

^b, g^ 95% CI [-3.20, -1.74]; p = 0.00; SDM = 2.0.
